# Machine learning for the prediction of in-hospital mortality in patients with spontaneous intracerebral hemorrhage in intensive care unit

**DOI:** 10.1038/s41598-024-65128-8

**Published:** 2024-06-20

**Authors:** Baojie Mao, Lichao Ling, Yuhang Pan, Rui Zhang, Wanning Zheng, Yanfei Shen, Wei Lu, Yuning Lu, Shanhu Xu, Jiong Wu, Ming Wang, Shu Wan

**Affiliations:** 1 Brain center, Affiliated Zhejiang Hospital, Zhejiang University School of Medicine, 1229 Gudun Road, Hangzhou, 310030 China; 2 The Second School of Clinical Medicine, Zhejiang Chinese Medical University, Hangzhou, 310053 China; 3 Urology Department, Lin’an Hospital of Traditional Chinese Medicine, Hangzhou, 311321 China; 4 Department of Intensive Care, Affiliated Zhejiang Hospital, Zhejiang University School of Medicine, Hangzhou, 310030 China; 5 ArteryFlow Technology Co., Ltd., Hangzhou, 310051 China

**Keywords:** Spontaneous intracerebral hemorrhage, Machine learning, Model prediction, Intensive care unit, MIMIC IV database, In-hospital mortality, Cerebrovascular disorders, Stroke

## Abstract

This study aimed to develop a machine learning (ML)-based tool for early and accurate prediction of in-hospital mortality risk in patients with spontaneous intracerebral hemorrhage (sICH) in the intensive care unit (ICU). We did a retrospective study in our study and identified cases of sICH from the MIMIC IV (n = 1486) and Zhejiang Hospital databases (n = 110). The model was constructed using features selected through LASSO regression. Among five well-known models, the selection of the best model was based on the area under the curve (AUC) in the validation cohort. We further analyzed calibration and decision curves to assess prediction results and visualized the impact of each variable on the model through SHapley Additive exPlanations. To facilitate accessibility, we also created a visual online calculation page for the model. The XGBoost exhibited high accuracy in both internal validation (AUC = 0.907) and external validation (AUC = 0.787) sets. Calibration curve and decision curve analyses showed that the model had no significant bias as well as being useful for supporting clinical decisions. XGBoost is an effective algorithm for predicting in-hospital mortality in patients with sICH, indicating its potential significance in the development of early warning systems.

## Introduction

Spontaneous intracerebral hemorrhage (sICH) is a critical neurological event characterized by bleeding within the intracerebral parenchyma, leading to a sudden and potentially life-threatening medical emergency^1^. Despite notable advancements in medical care, the overall prognosis of sICH remains poor, primarily attributed to inflammatory responses, oxidative stress, and other mechanisms, resulting in a significant proportion of patients succumbing during hospitalization^2,3^. Accurate prediction of in-hospital mortality holds paramount importance in guiding clinical decision-making and optimizing resource allocation. Therefore, there is an urgent need for an advanced model that can help predict the prognosis of patients with sICH.

In recent years, there has been a growing interest in the application of machine learning (ML) techniques to investigate various clinical diseases^4–8^. Compared to traditional statistics, ML exhibits characteristics such as handling complex nonlinear data, offering high flexibility, and enabling continuous learning and improvement^9,10^. Prior attempts have been made in the field of sICH^11,12^. However, there is still a pressing need for models that can accurately predict severe sICH cases in ICU.

The interpretability of machine learning models is crucial for enhancing trust among healthcare professionals. Therefore, Shapley Additive exPlanations (SHAP), a game-theoretic technique pioneered by Lundberg and Lee, effectively mitigates the black-box nature of ML models by offering consistent interpretability. This powerful method has been successfully applied in various contexts, including predicting the prognosis of patients with non-traumatic subarachnoid hemorrhage^13^, forecasting the onset of acute kidney injury following cardiac surgery^14^, and anticipating the development of sepsis in patients with COVID-19^15^.In this study, our objective is to develop and validate aML-based predictive model for in-hospital mortality among sICH patients. By utilizing a comprehensive set of clinical and demographic features, we aim to provide clinicians with a robust tool to accurately assess the risk of mortality in real-time, thus facilitating timely interventions and improving patient care.

## Methods

### Database and ethics

The Medical Information Mart for Intensive Care-IV (MIMIC-IV) is an open and freely accessible critical care database that contains comprehensive clinical data of patients admitted to a tertiary academic medical center in Boston, MA, USA, from 2008 to 2019. The database includes essential patient information, vital signs, laboratory indicators, treatment details, and survival data. The usage of data from MIMIC-IV has been granted approval by the Institutional Review Boards of Beth Israel Deaconess Medical Center (Boston, MA) and Massachusetts Institute of Technology (MIT; Cambridge, MA). As all personal data in this database is encrypted, informed consent was waived. One of the authors (Mao, Baojie) obtained access to the database and was responsible for data extraction (certification number 46148427). In addition, we recruited patients with cerebral hemorrhage who were admitted to ICU from December 2018 to February 2023 in Zhejiang Hospital. The study protocol was approved by the Ethics Review Committee of Zhejiang Hospital (No. 2023 Pro-examination (58 K)). All methods and procedures were carried out in accordance with the Declaration of Helsinki. All patient data were anonymized. No patient-identifiable data were recorded throughout the study. Given that this study was purely observational, written consent from patients was not required.

### Data extraction and outcomes

Clinical and laboratory variables were meticulously collected within 24 h of admission to the Intensive Care Unit (ICU). In the case of variables with multiple measurements, mean values were calculated and utilized for analysis. A total of forty-six variables were included in the data collection process. These encompassed patient characteristics (age, gender), vital signs (respiratory rate, blood pressure, heart rate, oxygen saturation, and temperature), laboratory data (routine blood analysis, renal function, coagulation, and blood gases), as well as comorbidities identified based on recorded International Classification of Diseases ICD-9 and ICD-10 codes. The comorbidities considered were hypertension, diabetes mellitus, chronic obstructive pulmonary disease (COPD), congestive heart failure, renal disease, liver disease, and malignancy. Furthermore, information regarding the usage of anticoagulant and vasoactive drugs, surgical status, Glasgow Coma Scale (GCS), Sequential Organ Failure Assessment (SOFA) scores, mechanical ventilation, and renal replacement therapy (RRT) was gathered. Due to the limited number of patients with missing data, we opted to exclude them from the analysis rather than attempting to estimate the missing values. The primary endpoint was all-cause in-hospital mortality.

### Cohort selection

1. Patients must be admitted to the ICU for the first time.
2. Patients must have a confirmed diagnosis of sICH.
3. Patients’ age should fall within the range of 18–90 years.
4. Patients must have an ICU length of stay exceeding 1 day.
5. Patients must have complete clinical data.

**Figure 1:**
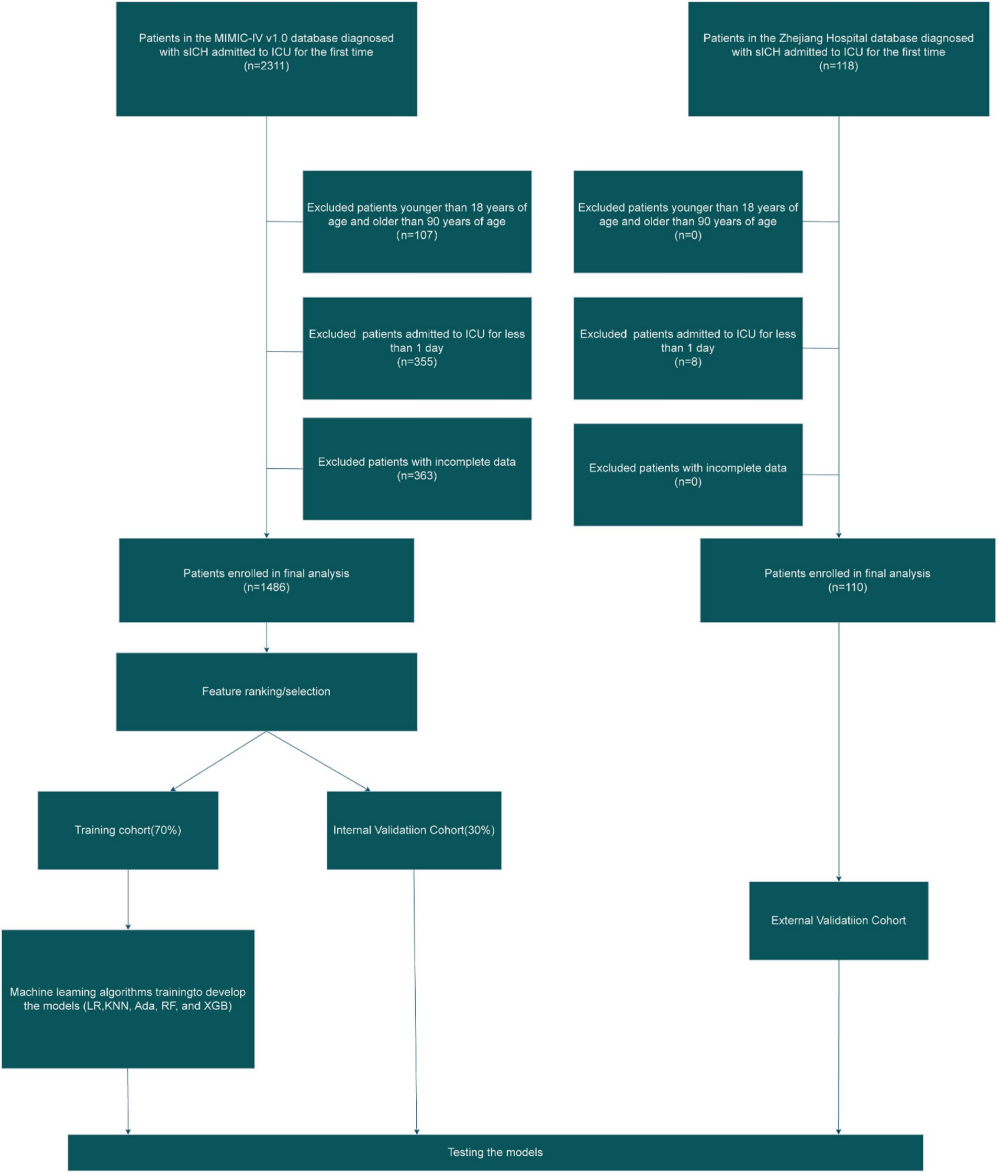
Model development process and flowchart of the study.

The flowchart for patient recruitment is shown in Fig. 1.

### Feature selection

We applied Lasso regression, a regularization technique, on the preprocessed dataset. Lasso performs feature selection by shrinking the coefficients of less important features to zero, effectively eliminating them from the model. The optimal regularization parameter (λ) for Lasso was determined using the coordinate descent algorithm. Following Lasso regression, the variables were ranked based on their corresponding non-zero coefficients. The final predictive model included the top 14 variables with the highest absolute coefficient values.

### Statistical analysis

The normality of the distribution was evaluated using the Kolmogorov–Smirnov test. Continuous variables were presented as mean with standard deviation if they followed a normal distribution, or as median with 25–75th percentile if they deviated from normality. The Student's t-test or Mann–Whitney test was applied accordingly to analyze the continuous variables. Categorical variables were presented as counts and percentages, and the chi-square test was utilized to compare the distributions.

In this study, we employed five different ML algorithms to develop models: Logistic regression (LR), K-nearest neighbors (KNN), Adaptive boosting (AdaBoost), Random forest (RF) and eXtreme Gradient Boosting algorithms (XGBoost). The MIMIC IV dataset was initially partitioned into a training set (70%) and an internal validation set (30%). Furthermore, we utilized the Zhejiang Hospital dataset as an external validation set. The validation process employed a bootstrap resampling technique with 1000 iterations to evaluate the model's performance. The area under the curve (AUC) and 95% confidence intervals (CI) were calculated. Furthermore, several evaluation metrics, including accuracy, sensitivity, specificity, Youden index, and F1 score, were computed. The performance of the model is assessed by conducting tenfold cross-validation and obtaining the average value. For hyperparameter selection, grid search methods were utilized.

To assess the performance and clinical applicability of the predictive model, we generated calibration curves and clinical decision curves. Calibration curves were used to evaluate the predictive accuracy and calibration of the model by comparing the predicted probabilities with actual observations. On the other hand, clinical decision curves were employed to determine the model's sensitivity and specificity at various decision thresholds, thus optimizing its predictive performance for clinical decision-making. After selecting the optimal model, we utilized the SHAP package in Python to demonstrate the importance of each feature. Subsequently, we developed a web-based visual interface using Streamlit to demonstrate the functionality of the selected machine learning model. Users can input relevant data parameters or upload datasets for real-time model evaluation. The model processes the input data, generating predictive outcomes based on the underlying learning patterns.

Statistical significance was set at *P* < 0.05, and all tests were two-tailed. Statistical analyses were performed using R software (version 4.3.1) or Python software (version 3.11).

## Result

### Baseline characteristics

The present study involved a total of 1596 patients, including 1486 patients from the internal cohort extracted from the MIMIC-IV database and 110 patients from the external cohort extracted from the Zhejiang Hospital database. In the internal cohort, there were 349 in-hospital deaths (23.48%), whereas the external cohort had 18 in-hospital deaths (16.36%). Table 1 provides an overview of the baseline characteristics for both the internal and external cohorts.

**Table 1:** Demographic and Clinical Characteristics of Hospitalization Survival and Mortality Groups in MIMIC IV and Zhejiang Hospital Database.

Characteristics	Survivors (n = 1137)	Non-survivors (n = 349)	*P*-value	Survivors (n = 92)	Non-survivors (n = 18)	*P*-value
Demographics						
Age, year, median (IQR)	66.00(55.00,76.00)	70.00 (59.00, 80.00)	< 0.001	54.00 (46.75, 62.25)	60.50 (51.25, 74.75)	0.055
Gender, male, n (%)	642 (56.46)	185 (53.01)	0.282	64 (69.57)	12 (66.67)	1.000
Comorbidities, n (%)						
Hyperlipidemia	422 (37.12)	125 (35.82)	0.707	0 (0.00)	0 (0.00)	NA
Diabetes	277 (24.36)	110 (31.52)	0.010	21 (22.83)	2 (11.11)	0.423
Hypertension	348 (30.61)	104 (29.80)	0.826	74 (80.43)	16 (88.89)	0.606
COPD	43 (3.78)	17 (4.87)	0.454	1 (1.09)	9 (50.00)	< 0.001
Congestive heart failure	115 (10.11)	57 (16.33)	0.002	1 (1.09)	9 (50.00)	1.000
Renal disease	229 (20.14)	133 (38.11)	< 0.001	4 (4.35)	4 (22.22)	0.030
Liver disease	57 (5.01)	36 (10.32)	< 0.001	16 (17.39)	4 (22.22)	0.879
Malignancy	160 (14.07)	35 (10.03)	0.062	5 (5.43)	2 (11.11)	0.708
Vital signs,median (IQR)						
SBP, mmHg	131.09 (122.54, 138.77)	130.04 (120.96, 136.91)	0.057	156.50 (136.00, 174.25)	153.50 (142.50, 157.75)	0.547
DBP, mmHg	68.00 (60.65, 75.73)	63.63 (57.41, 71.29)	< 0.001	81.00 (75.00, 93.00)	68.00 (64.25, 79.75)	0.003
MBP, mmHg	85.76 (78.60, 92.09)	82.09 (75.52, 88.93)	< 0.001	105.50 (95.75, 118.00)	97.50 (87.67, 104.58)	0.033
Temperature, °C	36.95 (36.75, 37.25)	37.12 (36.72, 37.53)	< 0.001	36.80 (36.60, 37.50)	37.25 (36.52, 37.95)	0.355
Heat rate, beats/min	79.04 (70.48, 88.16)	82.96 (74.36, 94.12)	< 0.001	83.50 (71.75, 90.00)	87.00 (63.50, 97.25)	0.497
Respiratory rate, beats/min	18.17 (16.44, 20.24)	18.85 (17.09, 21.27)	< 0.001	17.00 (15.75, 18.00)	16.00 (14.25, 18.00)	0.696
Blood oxygen saturation, %	97.22 (96.00, 98.60)	98.34 (96.91, 99.27)	< 0.001	100.00 (99.00, 100.00)	92.50 (90.62, 94.00)	< 0.001
Laboratory findings, median (IQR)						
WBC, 109/L	9.90 (7.90, 12.90)	11.90 (9.57, 14.93)	< 0.001	11.62 (9.55,14.10)	10.93 (8.84, 14.60)	0.929
RBC, 109/L	4.05 (3.58, 4.42)	3.82 (3.37, 4.27)	< 0.001	3.99 (3.54, 4.39)	3.87 (3.31, 4.65)	0.616
Platelet, 109/L	205.00 (165.50, 258.00)	189.00 (141.00, 240.00)	< 0.001	188.25 (147.38, 233.62)	164.00 (130.38, 232.62)	0.529
RDW, %	13.80 (13.10, 14.75)	14.15 (13.50, 15.60)	< 0.001	13.00(12.40, 13.60)	13.47 (13.00, 14.19)	0.017
Hematocrit, %	36.50 (32.70, 39.80)	34.90 (30.77, 38.70)	< 0.001	36.35 (33.11, 39.82)	36.20 (30.32, 41.48)	0.695
Hemoglobin, g/dL	12.17 (10.90, 13.30)	11.60 (10.00,13.00)	< 0.001	12.25 (10.70,13.43)	12.22 (10.25, 13.68)	0.731
BUN, mg/Dl	15.00 (11.50, 20.50)	18.50 (13.50, 28.00)	< 0.001	4.65 (3.70, 6.29)	6.30 (5.21, 10.31)	0.002
Creatinine, mg/dL	0.85 (0.70, 1.10)	1.00 (0.75, 1.50)	< 0.001	0.81 (0.65, 1.01)	0.98 (0.65, 1.34)	0.212
Glucose, mg/dL	127.00 (108.00, 151.00)	148.50 (124.00, 183.75)	< 0.001	75.88 (65.35, 94.08)	95.70 (81.00, 103.08)	0.009
Calcium, mmol/L	8.70 (8.35, 9.10)	8.55 (8.18, 9.00)	0.001	8.37 (8.16, 8.68)	8.56 (8.27, 8.67)	0.383
Potassium, mmol/L	3.90 (3.60, 4.20)	3.97 (3.70, 4.30)	0.004	3.74 (3.49, 3.98)	4.00 (3.56, 4.13)	0.117
Sodium, mmol/L	140.00 (137.50, 142.00)	140.50 (138.00, 144.00)	< 0.001	139.68 (138.10, 141.93)	139.86 (138.49, 143.58)	0.648
Chloride, mmol/L	104.00 (101.50, 107.00)	105.00 (101.00, 109.50)	0.002	105.35 (103.50, 107.93)	106.70 (103.53, 108.07)	0.455
MCH, pg	30.25 (28.90, 31.60)	30.50 (28.95, 31.75)	0.236	30.70 (29.64, 31.72)	30.70 (29.64, 31.72)	0.862
MCHC, g/dL	33.30 (32.35, 34.30)	33.10 (32.17, 34.03)	0.025	33.50 (32.94, 34.00)	33.47 (32.80, 33.84)	0.799
MCV, fl	91.00 (87.00, 94.00)	92.00 (88.00, 96.00)	0.002	91.50 (90.20, 93.40)	91.30 (89.82, 93.20)	0.677
INR, s	1.10 (1.10, 1.20)	1.20 (1.10, 1.30)	< 0.001	1.07 (1.02, 1.13)	1.11 (1.07, 1.18)	0.053
PT, s	12.50 (11.60, 13.80)	12.95 (12.00, 14.40)	< 0.001	13.90 (13.30, 14.40)	14.45 (13.46, 15.42)	0.041
PTT, s	27.90 (25.60, 30.90)	28.30 (25.35, 31.20)	0.399	35.10 (32.54, 38.32)	40.20 (36.16, 44.65)	0.004
Anion gap, mmol/L	14.00 (13.00, 16.00)	15.00 (13.00, 17.33)	< 0.001	9.15 (7.39, 10.40)	7.70 (6.17, 9.38)	0.076
Bicarbonate, mmol/L	23.50 (21.33, 25.50)	23.00 (20.33, 25.00)	< 0.001	22.41 (20.81, 23.80)	20.45 (19.47, 23.17)	0.196
Prognostic scoring system, median (IQR)						
SOFA	3.00 (2.00, 5.00)	6.00 (4.00, 8.00)	< 0.001	3.00 (2.00, 4.00)	9.00 (7.00, 10.00)	< 0.001
GCS	12.00 (8.00, 14.00)	7.00 (4.00, 15.00)	< 0.001	8.00 (5.00, 11.00)	4.00 (3.00, 5.00)	< 0.001
Treatment information, n (%)						
Mechanical ventilation	854 (75.11)	329 (94.27)	< 0.001	91 (98.91)	18 (100.00)	1.000
RRT	29 (2.55)	26 (7.45)	< 0.001	0 (0.00)	2 (11.11)	0.024
Use of mannitol	164 (14.42)	134 (38.40)	< 0.001	82 (89.13)	14 (77.78)	0.350
Use of anticoagulants	996 (87.60)	213 (61.03)	< 0.001	14 (15.22)	5 (27.78)	0.343
Use of vasoactive drugs	33 (2.90)	58 (16.62)	< 0.001	24 (26.09)	14 (77.78)	< 0.001
Surgical intervention	136 (11.96)	30 (8.60)	0.099	90 (97.83)	16 (88.89)	0.244

*COPD* chronic obstructive pulmonary disease, *SBP* systolic blood pressure, *DBP* diastolic blood pressure, *MBP* mean blood pressure, *WBC* white blood cell, *RBC* red blood cell, *RDW* red blood cell distribution width, *BUN* blood urea nitrogen, *MCH* mean corpuscular hemoglobin, *MCHC* mean corpuscular hemoglobin concentration, *MCV* mean corpuscular volume, *INR* international normalized, *PT* prothrombin time, *PTT* partial thromboplastin time, ratio, *SOFA* sequential organ failure assessment, *GCS* Glasgow Coma Scale, *RRT* renal replacement therapy.

### Key variables

**Figure 2:**
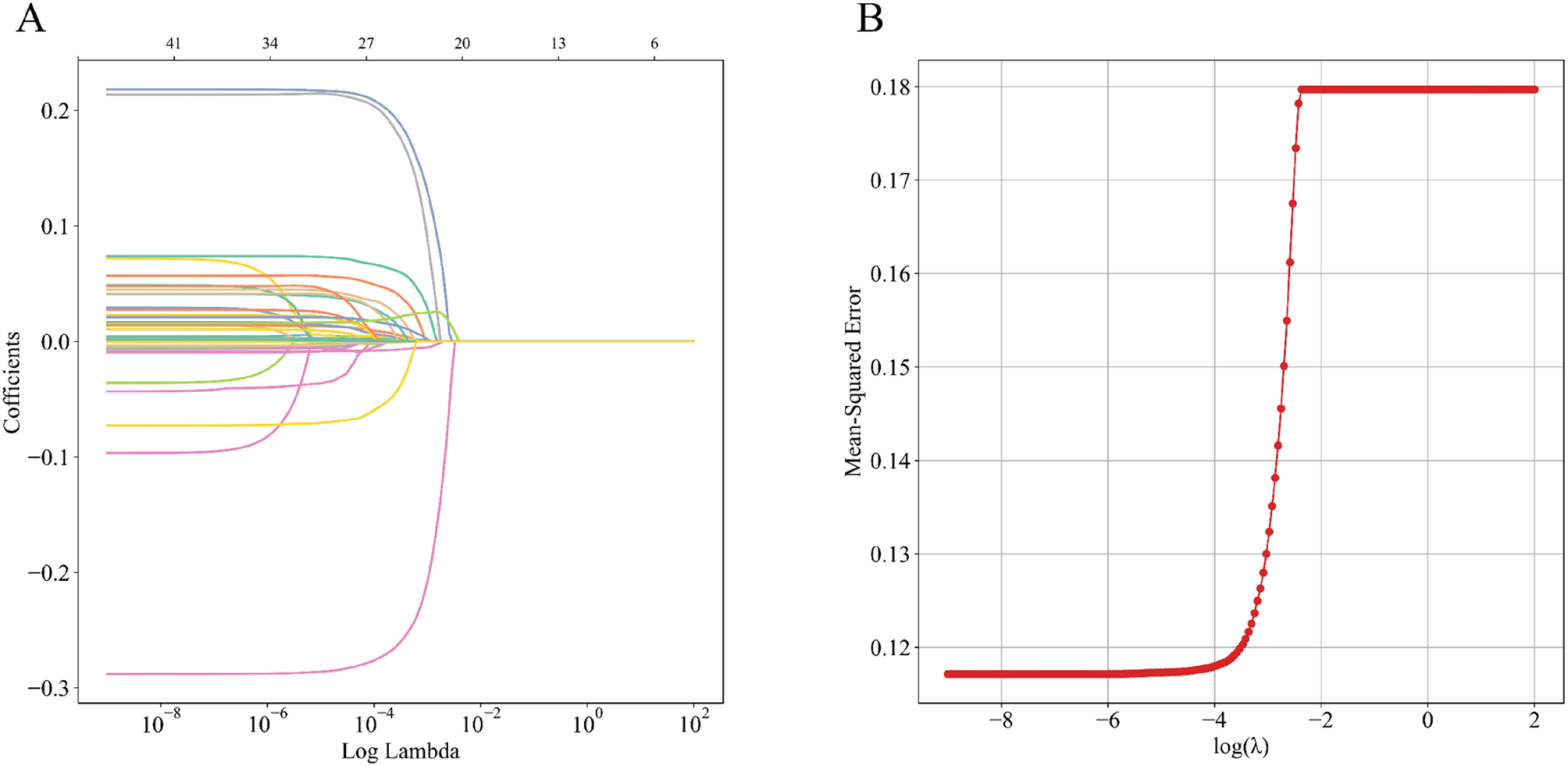
Demographic and clinical feature selection. The automated feature selection process for 46 clinical factors was executed utilizing the LASSO, aiming to minimize the binomial deviance loss function, shrink coefficients, and generate some zero coefficients to facilitate efficient feature selection (**A**). Subsequently, the algorithm identified and retained 14 filtered features with non-zero coefficients for integration into the model generation process (**B**).

Within the training set, LASSO regression was applied for automated feature selection as illustrated in Fig. 2. Lasso regression is a method for regression analysis that reduces unnecessary model complexity by introducing a regularization term (λ) for variable selection and complexity adjustment. From the initial pool of 46 candidate variables, we identified the top 14 based on their importance and integrated them into the final model. The selected variables encompassed: use of anticoagulants, use of mannitol, use of vasoactive drugs, mechanical ventilation, temperature, surgical intervention, serum potassium, heart failure, blood oxygen saturation, SOFA, GCS, serum sodium, RDW and serum chloride.

### Model performance

**Figure 3:**
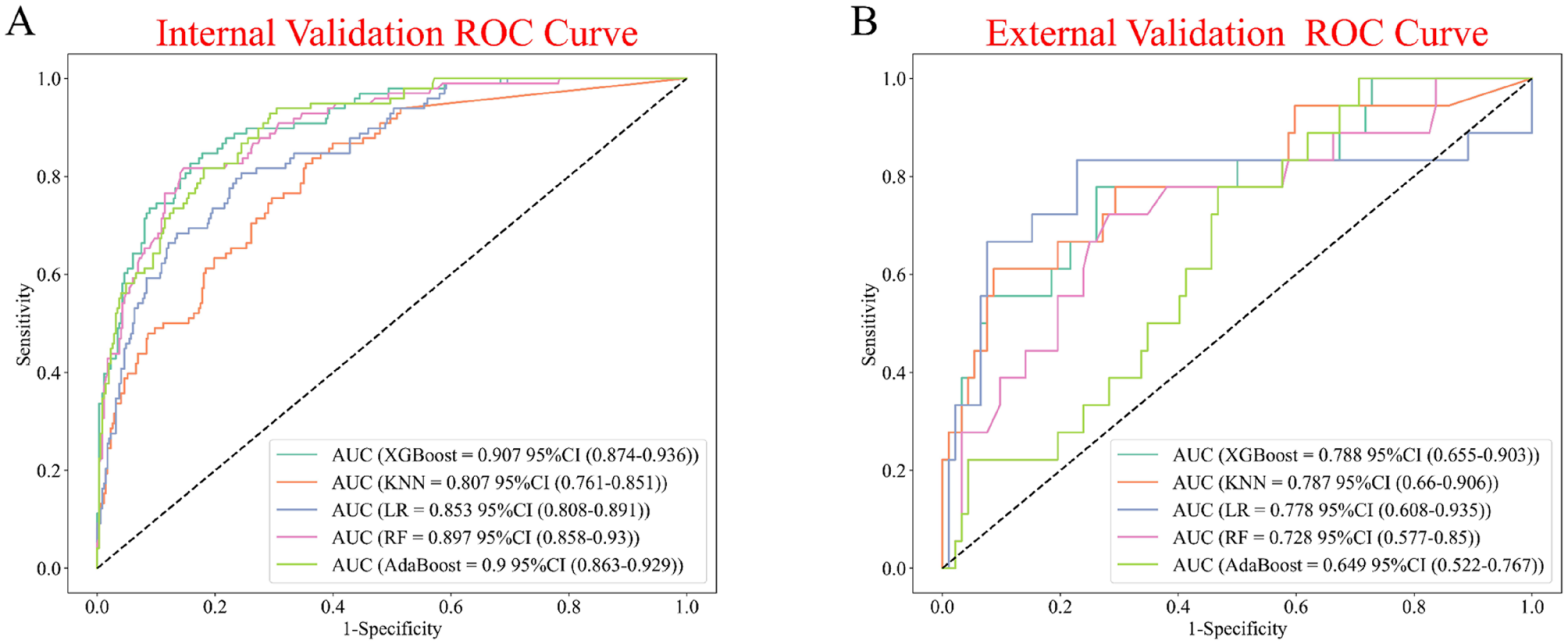
Area under the receiver operating characteristic curve for machine learning models in the internal validation queue (**A**) and external validation queue (**B**). *ROC* receiver operate characteristics, *CI* confidence intervals.

The discriminative ability of all models to predict mortality is shown in Fig. 3 and Table 2. In the training set, XGBoost, KNN, LR, RF, and AdaBoost models were built, and the AUCs of the internal validation set were 0. 907, 0.808, 0.851, 0.897, and 0.900, respectively. Note that the prediction performance of the XGBOOST model was the highest among these five models (AUC 0.907; 95% CI 0.875–0.939; accuracy: 0.874; sensitivity: 0.582). In the external validation set, the XGBoost model demonstrated predictive power with an AUC of 0.788, second only to the LR model, which achieved an AUC of 0.790.

**Table 2:** Predictive performance of machine learning models in internal and external validation sets.

Model	AUC	Accuracy	Sensitivity	Specificity	Youden index	F1 score
Internal validation cohort						
XGBoost	0.907	0.874	0.582	0.957	0.769	0.671
KNN	0.807	0.823	0.337	0.960	0.648	0.445
Logistic regression	0.851	0.843	0.551	0.925	0.738	0.606
Random forest	0.897	0.858	0.602	0.931	0.767	0.652
AdaBoost	0.900	0.865	0.561	0.951	0.756	0.647
External validation cohort						
XGBoost	0.788	0.554	0.833	0.500	0.667	0.380
KNN	0.787	0.855	0.500	0.924	0.712	0.529
Logistic regression	0.790	0.436	0.833	0.359	0.596	0.326
Random forest	0.728	0.436	0.833	0.359	0.596	0.326
AdaBoost	0.650	0.500	0.778	0.446	0.612	0.337

**Figure 4:**
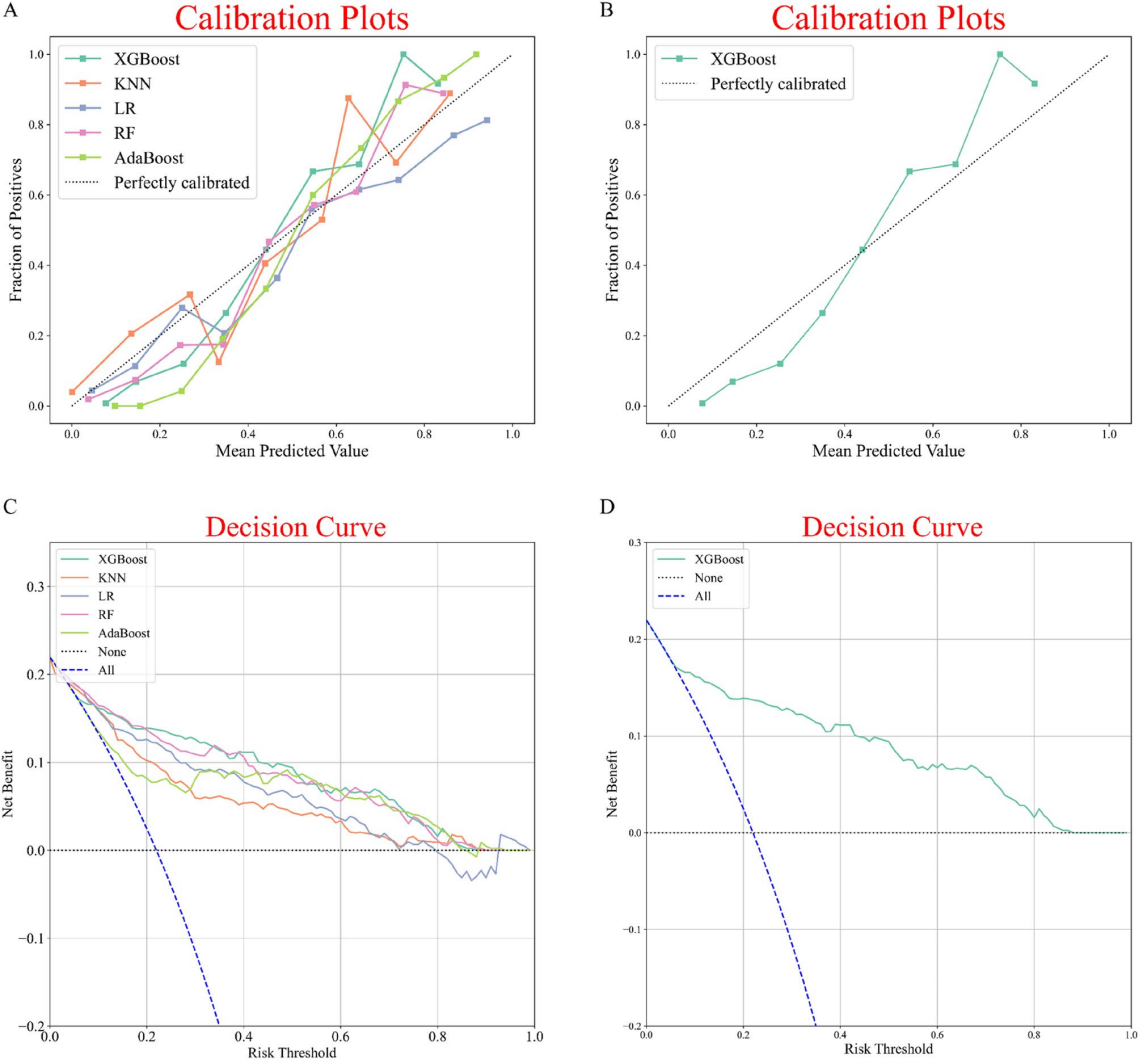
Calibration plots of five ML models in the internal validation queue (**A**,**B**). Decision curve analysis for five ML models in the internal validation queue (**C**,**D**).

Figure 4A shows the calibration plots for all five models. The calibration curve analysis showed that XGBoost was accurately calibrated in predicting the risk of in-hospital death, with no significant over or underestimation (Fig. 4B). In addition, the Decision Curve Analysis (DCA) for XGBoost has the highest net benefit across risk thresholds compared to all other models, as shown in Fig. 4C,D.

**Figure 5:**
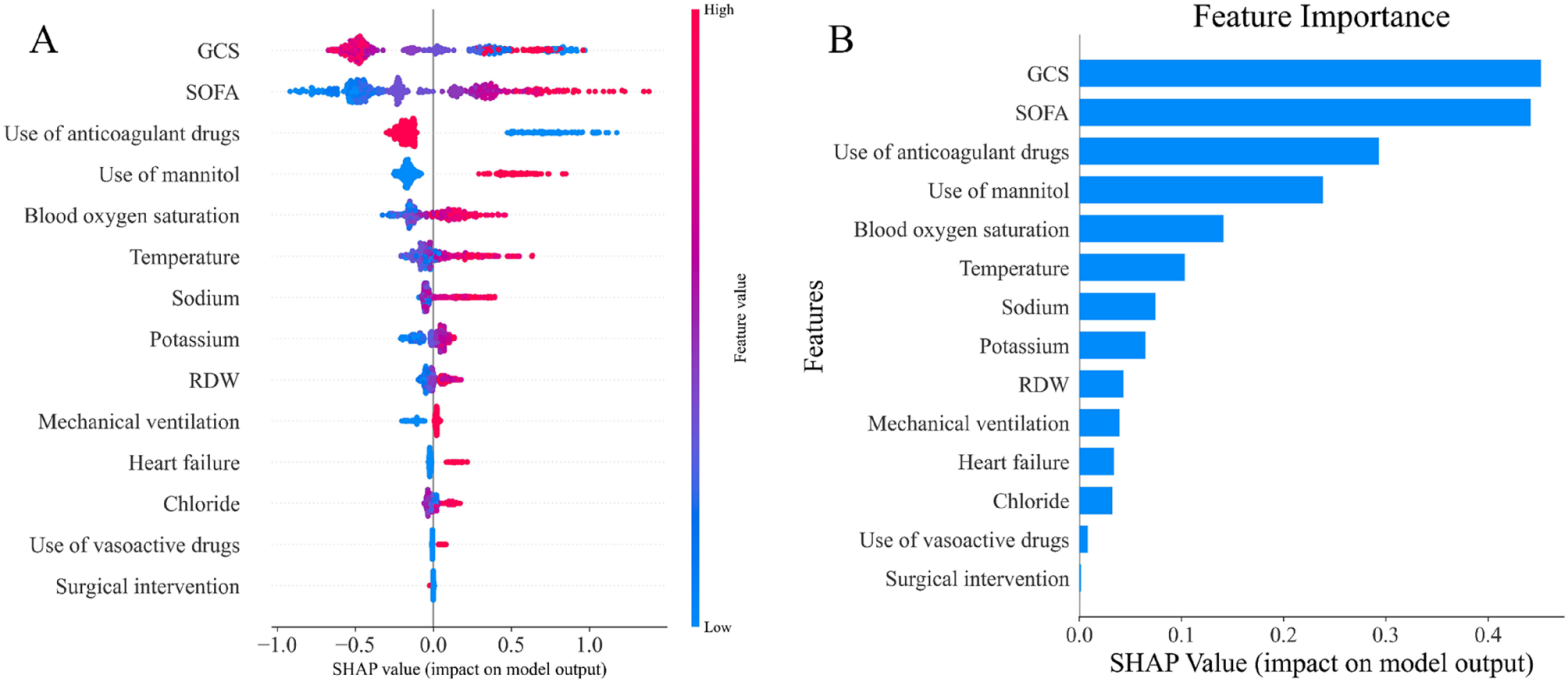
Scatter plot of variables for SHAP analysis (**A**) and importance ranking plot (**B**) of the XGBoost. A visual representation illustrates the importance of each feature in the XGBoost model, depicting the relationship between them. The color scale indicates the variable values, with red denoting higher values and blue indicating lower values.

The importance of features derived from XGBoost model is shown in Fig. 5. GCS score was the most influential feature followed by SOFA score, use of anticoagulants, use of mannitol, oxygen saturation, body temperature, serum sodium, serum potassium, RDW, mechanical ventilation, heart failure, serum chloride, use of vasoactive drugs and surgical intervention.

### Application of the model

**Figure 6:**
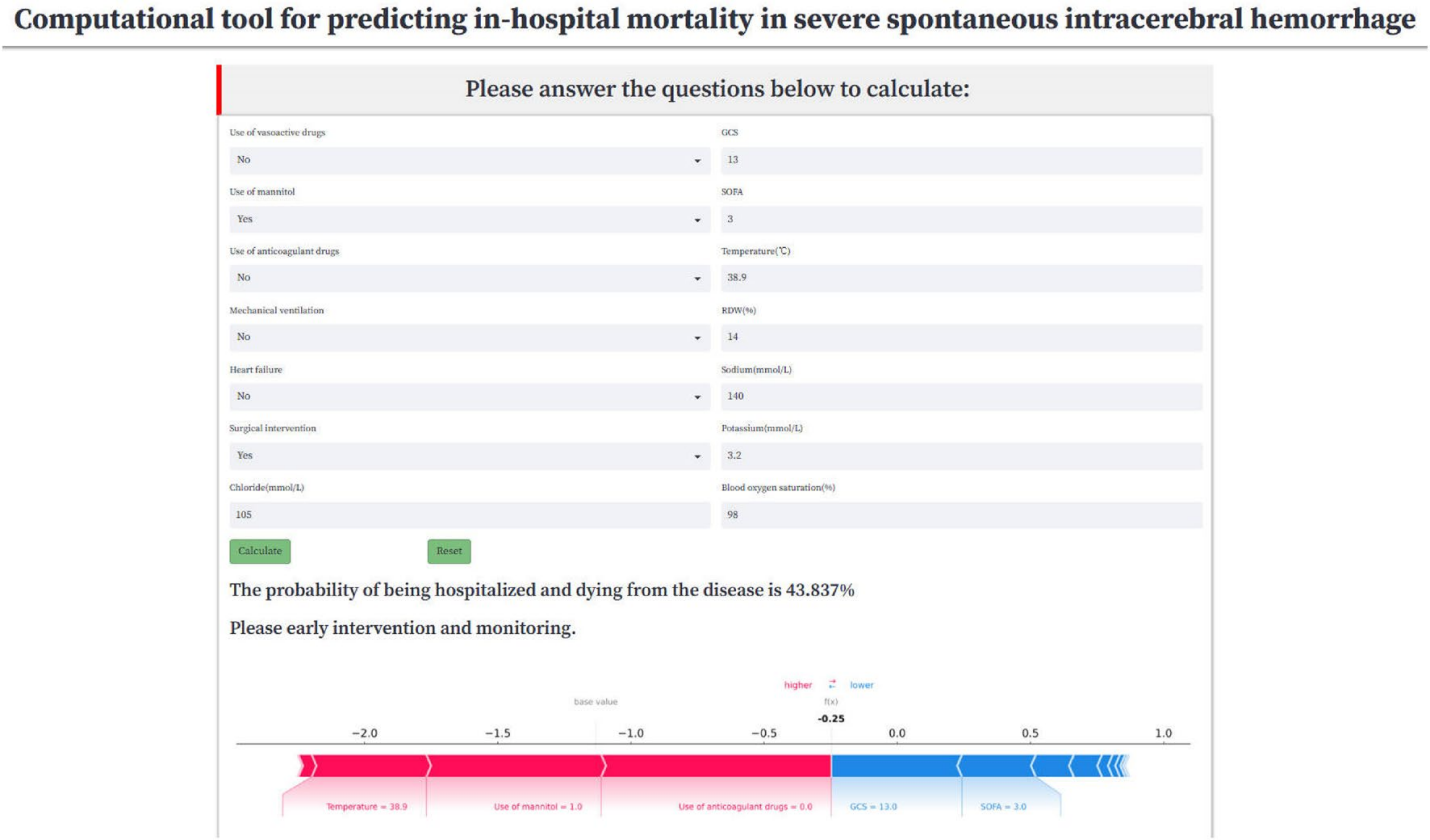
Case of website usage. Enter input values to determine the prognosis for sICH, and show the contribution of the variable shap value to the prediction. An in-hospital mortality rate of 44.812% was predicted. Additionally, factors including body temperature, non-utilization of anticoagulants, and mannitol usage were linked to a unfavorable prognosis in patients with sICH.

Additionally, a web-based computational tool using the XGBoost algorithm model has been developed to enable clinicians in real-time prediction of the prognosis for patients with severe sICH. (accessible at https://sich-mimic.streamlit.app/). Figure 6 shows an example of using a real-time prediction tool for web pages. This example highlights the patient's heightened risk of in-hospital mortality and indicates that variables such as temperature, medications, and other factors serve as prognostic risk factors. Clinicians are advised to promptly regulate the patient's temperature, consider conservative anticoagulant therapy, and evaluate the discontinuation of mannitol if deemed feasible and suitable.

## Discussion

In this retrospective study, we developed and validated a clinical feature-based machine learning algorithm for predicting in-hospital mortality in critically ill patients with sICH.Among the tested models, the XGBOOST model demonstrated the highest prediction performance. Through advanced machine learning techniques, we successfully identified several key clinical features strongly associated with in-hospital mortality, including GCS score, SOFA score, use of mannitol medication, use of anticoagulant medication, vital signs, serum electrolytes, RDW, among others. These findings are significant and warrant further investigation. Additionally, we have created an easy-to-use web-based calculator to assist clinicians in making informed decisions regarding further treatment.

Among various types of strokes, cerebral hemorrhage is characterized by a relatively high in-hospital mortality rate, especially in patients admitted to ICU. The in-hospital mortality rate of patients varies based on both the location and volume of the hematoma. Previous studies have reported an early mortality rate of 40% and a long-term mortality rate as high as 60% for sICH patients^16–18^.Marika Fallenius et al. analyzed patients admitted to the ICU with severe cerebral hemorrhage and found a mortality rate of 42% for supratentorial sICH patients and 49% for infratentorial sICH patients^19^. Additionally, researchers investigated a 30-day mortality rate of up to 54% for patients with severe sICH in the southern region of Spain, and this rate increased to 60% for patients with hematoma volumes exceeding 30 ml^20^. The mortality rate observed in our study was lower compared to the case-fatality rates reported in previous studies. This difference may be attributed to our exclusion of patients admitted for less than one day or those automatically discharged, as well as differences in medical conditions.

In this study, we employed five distinct ML methods to develop predictive models. The performance evaluation of these algorithms was based on six common metrics (AUC, F1 score, accuracy, sensitivity, specificity and Youden Index). Notably, the results unequivocally indicate that the XGBoost model exhibits the most superior performance and predictive stability, which contrasts with previous findings favoring the Random Forest model^21^. XGBoost is an efficient, flexible, and scalable ML algorithm, renowned for its classification capabilities. To mitigate overfitting and optimize its performance, XGBoost employs techniques such as improved subsampling rates, learning rates, and maximum tree depth control^22^. Zhu et al. evaluated data from ICU patients who were intubated due to respiratory failure and received mechanical ventilation. They utilized seven learning algorithms to predict in-hospital mortality, with XGBoost demonstrating the best overall performance^23^. Similarly, Hu et al. incorporated data from 8817 sepsis patients into seven models to predict in-hospital mortality, and they also found that the XGBoost model exhibited the most effective predictive ability^24^.

Despite the success of algorithms in this field, one of the current challenges lies in the need to interpret the “black box” of ML. Thus, we utilized the visualization function in SHAP to identify the impact of specific variable values on the model output. As anticipated, the GCS score takes the top position in the SHAP importance ranking. The GCS is a widely used scale for assessing the level of consciousness, with scores ranging from 3 to 15. Previous studies have consistently demonstrated the importance of the GCS score in evaluating the severity of neurological disorders^21,25,26^. The SOFA score serves as a valuable tool for quantifying the extent of organ dysfunction or failure at the point of ICU admission and has found widespread application in predicting in-hospital mortality in this setting^27–29^. It has been observed that the SOFA score exhibits superior predictive performance compared to other scoring systems when it comes to infection-related in-hospital mortality in ICU patients^30^. The use of anticoagulants in patients with cerebral hemorrhage and the timing of anticoagulant use remain controversial, and some studies have suggested that anticoagulants have a positive effect on patient prognosis. This might be because the use of anticoagulants in critically ill patients reduces complications such as thromboembolism and does not significantly increase bleeding complications^31^. Currently, the primary non-surgical treatment for cerebral hemorrhage involves the use of drugs like mannitol to reduce intracranial pressure. However, our study revealed that the use of mannitol may lead to a poor prognosis for patients with this disease. According to current guidelines, hypertonic saline demonstrates superior efficacy in managing cerebral edema associated with cerebral hemorrhage compared to mannitol^32^. Mannitol, which can elevate the risk of intracranial hemorrhage, may be less preferable in such cases. Oximetry ranked fifth in importance in our model. However, contrary to clinical expectations, we observed that oxygen saturation was lower in survivors compared to non-survivors in the internal cohort (97.22% vs. 98.34%) at baseline. It is possible that over-oxygenating ICU patients within normal oxygen saturation levels can lead to unfavorable prognoses and more adverse outcomes^33^. Moreover, prior research has established that electrolyte disturbances represent an independent risk factor for an unfavorable prognosis in stroke patients^34–36^. Lastly, to our surprise, the study indicates that in critically ill patients, surgical treatment may not hold significant importance. The low rate of surgery in sICH in the internal MIMIC dataset (8.60–11.96%) may be due to the fact that we included both critically and mildly ill patients, leading to a weakening of the influence of surgical intervention as an important factor, as it is known that surgical treatment may be more effective in patients with high bleeding volumes. This study holds significant clinical and methodological implications. Firstly, we implemented an external validation set to mitigate the risk of model overfitting. Secondly, the model was developed using readily available data collected within 24 h of patient admission, enabling early and accurate mortality prediction. This provides clinicians with more time to adjust treatment strategies accordingly. Thirdly, the study sheds light on previously overlooked factors, such as anticoagulant use and RDW, which are now identifiable. Integrating these factors with machine learning methods enhances the predictive performance significantly. Lastly, to facilitate bedside use by clinicians, a user-friendly calculator based on the model was developed.

## Limitation

However, our study has several limitations that need to be acknowledged. Firstly, it was a retrospective and observational study, which may introduce certain research biases. Secondly, as our study was focused on patients with sICH, we did not include information on radiologic variables, such as hematoma volume or location, which could potentially provide additional insights into the disease. Thirdly, the complexity of the model with 14 inputs may pose challenges for practical implementation in clinical settings, suggesting the necessity of integrating the algorithms with electronic medical record systems. Fourthly, variations in data collection methods between the open dataset and the local dataset may introduce realistic discrepancies. Lastly, the diversity of patient populations across different ICUs, as evidenced by differing death rates between hospitals, may impact the generalizability of the study findings, highlighting the importance of considering regional factors in result interpretation. It is possible that the local dataset represents a subgroup of critical care cases within the larger open dataset.

## Conclusion

The XGBoost model demonstrated superior performance in predicting short-term mortality among sICH patients. Our findings indicate that factors such as GCS, SOFA score, mannitol use, anticoagulant use, oxygen saturation, time of ICU admission, temperature, serum sodium, mechanical ventilation, and serum potassium are strongly associated with in-hospital mortality in sICH patients. This newly developed risk model is expected to serve as a convenient tool for risk stratification.

### Supplementary Information

Supplementary Information.

## Data Availability

The datasets and code used in the present study are available from the corresponding authors on reasonable request.

## Abbreviations

sICH: Spontaneous intracerebral hemorrhage
ICU: Intensive care unit
ML: Machine learning
LR: Logistic regression
KNN: K-nearest neighbors
AdaBoost: Adaptive boosting
RF: Random forest
XGBoost: eXtreme Gradient Boosting
AUC: Area under the curve
SHAP: SHapley Additive exPlanations
GCS: Glasgow Coma Scale
SOFA: Sequential Organ Failure Assessment
RDW: Red blood cell distribution width
MIMIC-IV: Medical Information Mart for Intensive Care-IV
COPD: Chronic obstructive pulmonary disease
RRT: Renal replacement therapy
CI: Confidence intervals
DCA: Decision curve analysis

## References

[1] Qureshi AI (2001). Spontaneous intracerebral hemorrhage. N. Engl. J. Med..

[2] Aronowski J, Zhao X (2011). Molecular pathophysiology of cerebral hemorrhage: Secondary brain injury. Stroke.

[3] Wang J (2010). Preclinical and clinical research on inflammation after intracerebral hemorrhage. Prog. Neurobiol..

[4] Esteva A (2017). Dermatologist-level classification of skin cancer with deep neural networks. Nature.

[5] Ambale-Venkatesh B (2017). Cardiovascular event prediction by machine learning: The multi-ethnic study of atherosclerosis. Circ. Res..

[6] Nomura A, Noguchi M, Kometani M, Furukawa K, Yoneda T (2021). Artificial intelligence in current diabetes management and prediction. Curr. Diab. Rep..

[7] Huang B (2021). Mortality prediction for patients with acute respiratory distress syndrome based on machine learning: A population-based study. Ann. Transl. Med..

[8] Huang T, Le D, Yuan L, Xu S, Peng X (2023). Machine learning for prediction of in-hospital mortality in lung cancer patients admitted to intensive care unit. PloS One.

[9] Raita Y (2019). Emergency department triage prediction of clinical outcomes using machine learning models. Crit. Care Lond. Engl..

[10] Manz CR (2020). Validation of a machine learning algorithm to predict 180-day mortality for outpatients with cancer. JAMA Oncol..

[11] Bunney G (2022). Predicting early seizures after intracerebral hemorrhage with machine learning. Neurocrit. Care.

[12] Rusche T (2023). Machine learning for onset prediction of patients with intracerebral hemorrhage. J. Clin. Med..

[13] Gu L (2024). Machine learning predictors of risk of death within 7 days in patients with non-traumatic subarachnoid hemorrhage in the intensive care unit: A multicenter retrospective study. Heliyon.

[14] Tseng P-Y (2020). Prediction of the development of acute kidney injury following cardiac surgery by machine learning. Crit. Care.

[15] Tang G (2021). Prediction of sepsis in COVID-19 using laboratory indicators. Front. Cell. Infect. Microbiol..

[16] Van Asch CJ (2010). Incidence, case fatality, and functional outcome of intracerebral haemorrhage over time, according to age, sex, and ethnic origin: A systematic review and meta-analysis. Lancet Neurol..

[17] Sacco S, Marini C, Toni D, Olivieri L, Carolei A (2009). Incidence and 10-year survival of intracerebral hemorrhage in a population-based registry. Stroke.

[18] Poon MTC, Fonville AF, Al-Shahi Salman R (2014). Long-term prognosis after intracerebral haemorrhage: Systematic review and meta-analysis. J. Neurol. Neurosurg. Psychiatry.

[19] Fallenius M (2019). Spontaneous intracerebral hemorrhage. Stroke.

[20] Rodríguez-Fernández S (2018). Validation of the ICH score in patients with spontaneous intracerebral haemorrhage admitted to the intensive care unit in Southern Spain. BMJ Open.

[21] Nie X (2020). Mortality prediction in cerebral hemorrhage patients using machine learning algorithms in intensive care units. Front. Neurol..

[22] Xu Y (2022). Predicting ICU mortality in rheumatic heart disease: Comparison of XGBoost and logistic regression. Front. Cardiovasc. Med..

[23] Zhu Y (2021). Machine learning prediction models for mechanically ventilated patients: Analyses of the MIMIC-III database. Front. Med..

[24] Hu C (2022). Interpretable machine learning for early prediction of prognosis in sepsis: A discovery and validation study. Infect. Dis. Ther..

[25] Teasdale G, Jennett B (1974). Assessment of coma and impaired consciousness. A practical scale. Lancet Lond. Engl..

[26] Agrawal N (2023). Comparison of admission GCS score to admission GCS-P and FOUR scores for prediction of outcomes among patients with traumatic brain injury in the intensive care unit in India. Acute Crit. Care.

[27] Ferreira FL (2001). Serial evaluation of the SOFA score to predict outcome in critically ill patients. JAMA.

[28] Vincent J-L (1996). The SOFA (Sepsis-related Organ Failure Assessment) score to describe organ dysfunction/failure: On behalf of the working group on sepsis-related problems of the European society of intensive care medicine (see contributors to the project in the appendix). Intensive Care Med..

[29] Cárdenas-Turanzas M (2012). Cross-validation of a Sequential Organ Failure Assessment score–based model to predict mortality in patients with cancer admitted to the intensive care unit. J. Crit. Care.

[30] Raith EP (2017). Prognostic accuracy of the SOFA score, SIRS criteria, and qSOFA score for in-hospital mortality among adults with suspected infection admitted to the intensive care unit. JAMA.

[31] Kuramatsu JB, Huttner HB (2019). Management of oral anticoagulation after intracerebral hemorrhage. Int. J. Stroke Off. J. Int. Stroke Soc..

[32] Cook AM (2020). Guidelines for the acute treatment of cerebral edema in neurocritical care patients. Neurocrit. Care.

[33] Barbateskovic M (2019). Higher versus lower fraction of inspired oxygen or targets of arterial oxygenation for adults admitted to the intensive care unit. Cochrane Database Syst. Rev..

[34] Bales J (2016). The effect of hyponatremia and sodium variability on outcomes in adults with aneurysmal subarachnoid hemorrhage. World Neurosurg..

[35] Matano F (2019). Serum glucose and potassium ratio as risk factors for cerebral vasospasm after aneurysmal subarachnoid hemorrhage. J. Stroke Cerebrovasc. Dis. Off. J. Natl. Stroke Assoc..

[36] Sadan O, Samuels O, Asbury WH, Hanfelt JJ, Singbartl K (2018). Low-chloride versus high-chloride hypertonic solution for the treatment of subarachnoid hemorrhage-related complications (The ACETatE trial): Study protocol for a pilot randomized controlled trial. Trials.

