# Supplementary material for: Machine learning for the prediction of in-hospital mortality in patients with spontaneous intracerebral hemorrhage in intensive care unit
Source: Sci Rep. 2024 Jun 20;14:14195. doi: 10.1038/s41598-024-65128-8 (PMC11190185; doi:10.1038/s41598-024-65128-8)
Supplement: Supplementary file 1 — Supplementary Information. [file 41598_2024_65128_MOESM1_ESM.docx]

**ICD codes for spontaneous cerebral hemorrhage**

| icd_code | icd_version |
| --- | --- |
| 431 | 9 |
| 4329 | 9 |
| I61 | 10 |
| I610 | 10 |
| I611 | 10 |
| I612 | 10 |
| I613 | 10 |
| I614 | 10 |
| I615 | 10 |
| I616 | 10 |
| I618 | 10 |
| I619 | 10 |
| I62 | 10 |
| I629 | 10 |

| Model | Hyperparameters dataset | target parameters |
| --- | --- | --- |
| XGBoost | 'learning_rate': [0.1, 0.01, 0.001],  'max_depth': [3, 5, 7],  'n_estimators': [100, 200, 300],  'subsample': [0.6, 0.8, 1.0],  'colsample_bytree': [0.6, 0.8, 1.0] | 'colsample_bytree': 0.6,  'learning_rate': 0.01,  'max_depth': 3,  'n_estimators': 300,  'subsample': 0.6 |
| KNN | 'n_neighbors': [3, 5, 7],  'weights': ['uniform', 'distance'],  'metric':['euclidean','manhattan'],  'leaf_size': [20, 30, 40],  'algorithm':['auto','ball_tree','kd_tree'] | 'algorithm': 'auto',  'leaf_size': 20,  'metric': 'manhattan',  'n_neighbors': 7,  'weights': 'distance' |
| Logistic | 'C': [1.0],  'max_iter': list(range(100, 1500, 200) | 'C': 1.0, 'max_iter': 700 |
| RandomForest | 'n_estimators':list(range(100,1500,200)),  'criterion': ['gini', 'entropy' | 'criterion': 'gini', 'n_estimators': 300 |
| Adaboost | 'n_estimators': [50, 100, 200],  'learning_rate': [0.1, 0.01, 0.001],  'base_estimator':[None, DecisionTreeClassifier(max_depth=1), DecisionTreeClassifier(max_depth=3)] | 'base_estimator':DecisionTreeClassifier(max_depth=3),  'learning_rate': 0.01,  'n_estimators': 200 |

**Datasets for model hyperparameter selection**
